# Pediatric Cancer Incidence, Temporal Trends, and Mortality in the United States by Health Disparities Indicators, SEER (1973–2014)

**DOI:** 10.3390/cancers17172848

**Published:** 2025-08-30

**Authors:** Prachi P. Chavan, Laurens Holmes

**Affiliations:** 1 Nemours/AIDHC Office of Health Equity and Inclusion, Wilmington, DE 19803, USA; 2 Department of Epidemiology, Biostatistics and Environmental Health, Joint School of Public Health, Old Dominion University, Norfolk, VA 23529, USA; 3 Master of Public Health Program, School of Health Professions, Joan & Macon Virginia Health Sciences, Eastern Virginia Medical School, Norfolk, VA 23510, USA; 4 Biological Sciences Department, University of Delaware, Newark, DE 19716, USA

**Keywords:** pediatric cancer trends, health disparities, cancer mortality, SEER, childhood cancer, epidemiology

## Abstract

Background: Pediatric cancer incidence has been increasing in the United States, despite improvement in pediatric cancer survival. This steady increase in incidence trends is not completely understood but maybe associated with social and environmental factors. In this study we aimed to assess the cumulative incidence, temporal trends, and mortality rates in pediatric cancer. Additionally, we examined sub-group variability in both incidence and mortality rates. Methods: Data from Surveillance, Epidemiology, and End Results (SEER) −18 from 1973–2014 were used for the purpose of analysis in this study. Age-adjusted incidence rates were used to assess temporal trends in cancer among children aged <1–19 years. Univariable and multivariable binomial regression models were used to examine the association between race and cancer mortality while adjusting for potential confounders. Results: There were 92,594 cancer diagnoses during this period. White children comprised 74,758, (80.7%), black children 10,030, (10.8%), and other races 6648, (7.2%). Overall the age-adjusted cumulative incidence was slightly higher among white children (16.4%) than black children (12.4%) and other (13.0%). Children aged 15–19 years and those in metropolitan regions were more likely to be diagnosed with pediatric cancer. Relative to females, males were 16% more likely to die from the disease [adjusted Risk Ratio (aRR): 1.16, 95% Confidence Interval (CI): 1.09–1.22]. Additionally, compared to white children, black children had higher mortality rates [(aRR): 1.37, 99% CI: 1.23–1.52]. Conclusions: There is an increasing trend in pediatric cancer incidence; while white children have the highest incidence, black children and males indicated a survival disadvantage, indicative of racial and sex variability in overall pediatric cancer in the United States.

## Simple Summary

Cancer remains the leading cause of pediatric mortality in the United States among children 0 to 19 years of age. The current study explored the temporal trends analyzing a large cohort of children with cancer diagnosis in USA using National Cancer Institute SEER data of four decades. The study design is a retrospective cohort using a multivariable binomial regression model following a probability distribution. While cumulative incidence of pediatric cancers remained higher among white children, a survival disadvantage was seen among black children. Regardless of race, age at diagnosis was higher among children aged 15 to 19 years. The understanding of these differences among biological and social factors requires further research on genetic epidemiology studies which can prevent pediatric malignancies and help advance existing treatment.

## 1. Introduction

Cancer, which is a genetic disease, remains one of the leading causes of disease-related mortality among children 0–14 years in the United States, albeit the improved survival [1,2] in some malignancies, mainly acute lymphocytic leukemia (ALL). The current increased mortality is due in part to advances in diagnostics and therapeutics. Despite the observed mortality rates especially in ALL, acute myeloid leukemia (AML) continues to present with challenges as well as sex and race variability in mortality rates. While pediatric cancer in the U.S. is rare, consisting of less than 1% of cancers diagnosed each year, incidences have increased over the years. Approximately 10,000 children are expected to be diagnosed with cancer in 2025 [3]. Between 1990 and 2004, 34,500 new pediatric cancer cases were reported in the United States and around 2004 the pediatric cancer mortalities reached 2223, of which leukemia was the most prevalent diagnosis of pediatric malignancy [4].

Leukemias are fast-growing cancers of the bone marrow and blood and represent the most diagnosed pediatric cancer [5]. One in three (33.3%) childhood cancer occurrences are leukemias, and three in four (75%) leukemia occurrences are ALL. Studies have shown that ALL peaks between 2–4 years of age and is more common among males and Hispanic children compared to other ethnicities [6,7,8]. However, AML is equally prevalent among both sexes and is the second most commonly occurring childhood leukemia [8].

The second most common type of pediatric cancer is cancer of the brain and central nervous system (CNS). The Brain/CNS tumor consists of abnormally growing tumors in both the brain and the spinal cord, and account for one in four childhood cancers (25%). Brain/CNS tumors are observed slightly more among males compared to females and about three in four children (75%) diagnosed with these malignant tumors survive for at least five years post-diagnosis, implying 75% relative survival [9,10]. Other common pediatric cancers include lymphoma, cancer of the lymph nodes, and renal cancer [11].

Unlike many adult cancers, pediatric cancers are often not related to lifestyle habits. Childhood cancers are usually associated with factors such as the environment, socio-demographic characteristics, and lack of access to proper preventive modalities [12]. While age, race, geography, and family history have been indicated as established risk factors in some adult malignancies, such as prostatic adenocarcinoma [13], pediatric malignancies lack this sort of characterization with respect to known risk factors. Mutations in human cells are not uncommon; however, mutations that lead to various pediatric cancers, and the factors that influence these results are not completely understood for children [14].

The current study examined the incidence, temporal trends, and mortality in pediatric cancer in United States based on health disparities indicators, namely race and sex. We hypothesized that (i) there is no difference in pediatric cancer incidence and temporal trends by race and sex in a representative sample of patients SEER (1973–2014), (ii) the mortality of pediatric cancer does not differ by race and sex, and (iii) racial differences in overall pediatric cancer incidence and mortality are not explained by racial and sex differences in geographical locale (SEER registries) and access to care (insurance).

## 2. Materials and Methods

### 2.1. Design

A population-based design, namely, a retrospective cohort, was used to examine the research questions that form the basis of the hypothesis in this study. This design is feasible given the preexisting data in SEER collected between 1973–2014.

### 2.2. Data Source

The data used for the study analysis were extracted from the Surveillance, Epidemiology, and End Results (SEER) Program of the National Cancer Institute’s (NCI) dataset from 1973–2014. SEER is a large cancer registry operated by the NCI. The registry began in 1973 with 9 SEER area termed registries and expanded in 1992 to include 4 more registries, which further expanded in 2005 to include 5 additional registries which makes the current registry in SEER at 18. The commonly identified registry from this enriched database includes 9, 11, 13, 17, and 18. The de-identified information in the SEER registry includes cancer patient diagnosis, socio demographics, tumor site and morphology, prognostic factors, treatment and follow-up for vital status, as well as some social drivers of health. Till date this is one of the most comprehensive population-based databases where the registry includes cancer diagnosis as well as cancer survivor information. The selection of the registries into The SEER program selects registries based on the available population-based data from the cancer centers [15].

The population characteristics of SEER participants is comparable to the population of the United States. While the participation of foreign-born participants in SEER is 17.9%, the participation of the United States population is 12.8%. Those with less than a high school diploma represents 16%, while those in the U.S. represent 14.6%. Additionally, those characterized below poverty level are equally represented in the U.S. population (14.1% vs. 14.3%).

### 2.3. Study Population

This study utilized the most recent SEER reports for cancer trends based on cancer diagnosis from year 1973–2014. The study population consisted of children <1 to 19 years of age, who were diagnosed with cancer from 1973–2014. The cancer diagnosis was classified based on International Classification of Disease (ICD-O-3) [16]. The total sample consisted of 92,594 participants, with white children representing 74,758 (80.7%), black children—10,030 (10.8%), other—6648 (7.2%), and 1158 were Unknown (1.2%). Cancer cases were categorized into four age groups: <1 years, 1–4 years, 5–9 years, 10–14 years, and 15–19 years. This study included cancer patients from the 18 SEER registries.

### 2.4. Study Variables

SEER registry collects information on socio-demographic characteristics of participants. We included age at diagnosis, sex, race, and geographic locale of participants for the purpose of this study. All socio-demographic characteristics included were self-reported and the variables were measured on a nominal scale (qualitative scale). The insurance variable was classified into any Medicare, insured, insured/no specifics, and uninsured. Location was categorized as rural, urban, and metropolitan.

### 2.5. Temporal Trends

The SEER Stat software was used to estimate the age-adjusted temporal trends in the pediatric age group of children <1 to 19 years [17]. The incidence and mortality rates for new primary cancer were expressed as per 100,000 persons at risk. The SEER registry data available through the year 2014 was used for analysis. In November of each year, SEER cases are reported to the NCI with approximately 98% completion rate for all sites of cancer, excluding tumors such as melanoma since they are incomplete [18]. The SEER cancer registries are updated regularly, and the SEER Stat software is used to address any delay in response rates. In this study, we demonstrated temporal trends for cumulative incidence and mortality rates for four racial groups [All Races; White; Black; Other (American Indian/Alaskan Native, and Asian/Pacific Islander)].

### 2.6. Power Estimation

The data in this study involved pre-existing cases of children diagnosed with cancer (n = 31,970). In order to detect a clinically meaningful difference between incidence and mortality, we estimated the power of the study using the following parameters: (i) Sample size (n = 31,970) by racial subgroups (White = 25,446), (Black = 3704), (Others = 2820). The sample size of the study was adequate to determine the racial heterogeneity in children with pediatric malignancies (power = 0.80) (ii) Effect size = 0.37, binomial regression risk ratio (37%) and (iii) Type I error tolerance and 95% C.I. (*p* < 0.05) for univariable model and 99% C.I. (*p* < 0.01) for multivariable model.

Based on these parameters, we estimated the power to be >80% (type II error tolerance <20%), which is sufficient power to detect a minimum difference of 10% to compare the mortality difference in children by race.

### 2.7. Statistical Analysis

The summary statistics which represented exploratory analysis was used to assess the distribution of patients by race, geography, sex, mortality, and insurance status. This process involved frequency and percentage of proportion as well as for nominal data such as sex.

To assess race as the function of mortality, we used univariable and multivariable binomial regression models. This model follows binomial probability distribution, which is appropriate when data are available in binary form or in proportion. To address for the confounding effect of social determinant variables such as health inequity in the medical insurance, we used a multivariable binomial regression model. In order for a potentially confounding variable to be entered into the model, we first assessed for the confounding effect of such a variable in the relationship between race and pediatric cancer mortality. This process involved Mantel–Haenszel stratification analysis, and considered a variable as confounding if the difference between the unadjusted and the stratified estimate is 10% or greater. We also examined variables for effect measure modification by considering the stratum specific point estimate and if there were substantial variability in the stratum specific estimate then such a variable was considered an effect measure modifier.

All tests were two-tailed, implying type I error tolerance (0.05/2). The type error tolerance was set as 95% confidence interval (alpha = 0.05) for the univariable model and 99% C.I. (alpha = 0.01) for the multivariable model. The entire analysis was performed using STATA (STATA corp, College Station, TX, USA).

## 3. Results

### 3.1. Distribution of Study Characteristics by Race

Table 1 demonstrates the study characteristics, namely, age, sex, insurance, geographic locale, mortality, first and second primary malignancy, and SEER registries stratified by race.

### 3.2. Cumulative Incidence

This study illustrates a representative sample of children diagnosed with cancer and followed for the disease, implying the cumulative incidence and mortality. Additionally, this sample assessed the incidence of pediatric malignancies and their mortalities by health disparities indicators, namely, race, sex, insurance, and geographic locale. The cumulative incidence rate based on the age-adjusted rates indicated a slightly higher incidence among white males (17.1%) compared to black males (16.6%). The incidence was also higher among white females (15.2%) compared to black females (12.2%).

### 3.3. Mortality

Table 1 illustrates the mortality implying survival in this sample and the variability of mortality by race, sex, and geography. Mortality distribution indicated black children (n = 684, 18.5%) with higher deaths compared to white children (n = 3312, 13.0%). Male children demonstrated higher mortality compared to females (n = 2540, 14.8% vs. n = 2052, 12.7%). The mortality was higher among children in rural areas (n = 52, 15.1%), intermediate in urban areas (n = 395, 14.7%), and lowest among children in metropolitan areas (n = 3979, 13.7%). Mortality varied by race, where black relative to white children indicated a higher mortality (Whites: 13.0%, Blacks: 18.5%). There were racial disparities in second primary malignancies as well. Compared to white children (2.0%), black children (2.1%) had a slightly higher incidence of second primary malignancies.

### 3.4. Sex

The cumulative incidence of the pediatric cancer indicated variability by sex as well as by race. Regardless of race the cumulative incidence was higher among males, specifically compared to black females, and black males had higher rates of pediatric malignancies (47.8% vs. 52.2%). The racial differences in males and females are demonstrated in Appendix A and Appendix B.

### 3.5. Geography

The pediatric malignancy in this SEER data indicated a pattern of cumulative incidence by geographic locale as well as by race. Regardless of race of the children, incidence was highest in metropolitan areas, intermediate in urban areas, and lowest in rural areas.

### 3.6. Age Group at Diagnosis

Regardless of race, the cumulative incidence by age at diagnosis was higher among age group 15–19 years, and intermediate among age group 1–4 years.

### 3.7. Insurance Coverage

There was racial variance in the access and utilization of care by the cancer patients as indicated by insurance. Black children compared to white children were more likely to be uninsured; likewise, black children compared to white children were more likely to have Medicaid (public insurance). In contrast, white children compared to black children were more likely to have private insurance (54.3% vs. 39.0%). Similarly, other races (American Indian/Alaska Native/Asian/Pacific Islander), which are difficult to characterize, also had the highest use of private insurance relative to black children.

### 3.8. Temporal Trends

**Figure 1:**
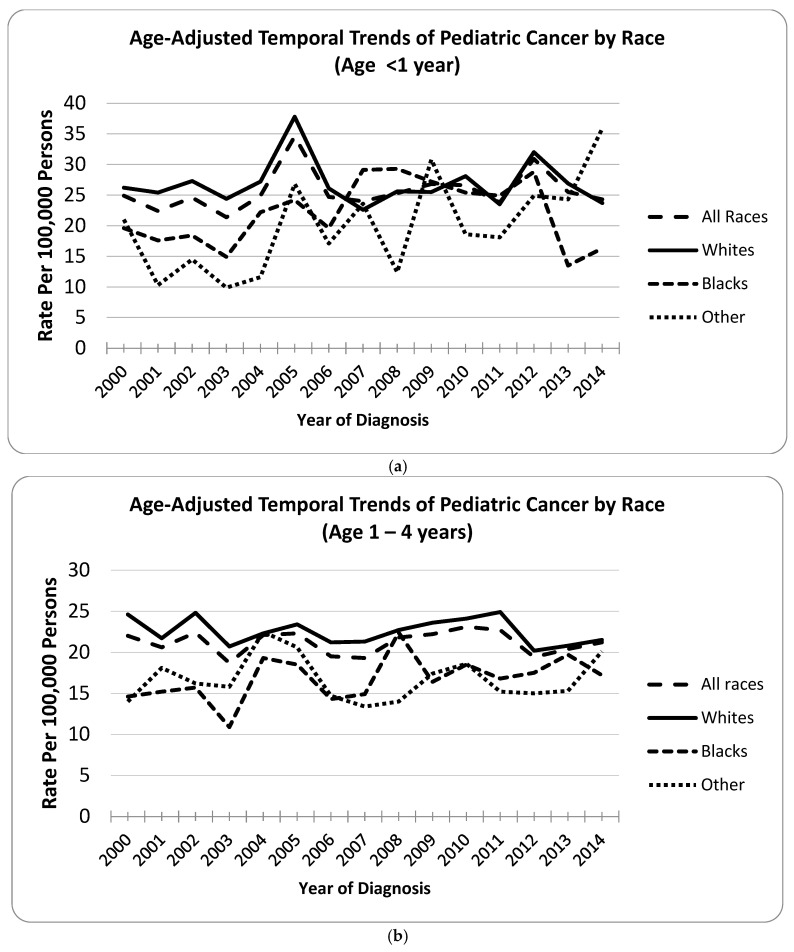

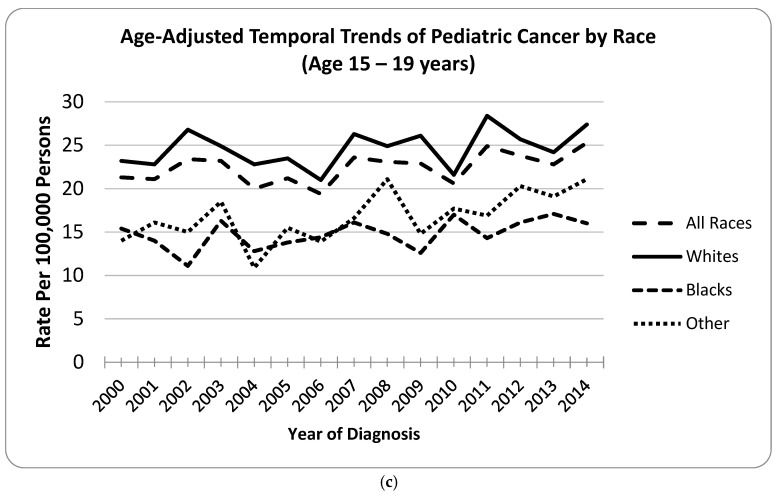
(**a**): Age-Adjusted Temporal Trends of Incidence in Pediatric Cancer by Race (age <1 year). (**b**): Age-Adjusted Temporal Trends of Incidence in Pediatric Cancer by Race (age 1–4 years). (**c**): Age-Adjusted Temporal Trends of Incidence in Pediatric Cancer by Race (Age 15–19 years).

Figure 1a illustrates the age at cancer temporal trend by race in children <1 year of age; the percent change was higher among white children, indicative of higher cumulative incidence. Figure 1b demonstrates age-adjusted temporal trends by 1–4 years of age at diagnosis. The cumulative incidence clearly indicated higher rates of tumor among white children despite the annual fluctuation between the years 2000–2014. Figure 1c shows the age-adjusted temporal trends in overall pediatric malignancy by age group 15–19 years. Despite the fluctuation in the annual incidence of pediatric tumor in this SEER sample, between 2000–2014, white children indicated a very clear increased pattern, indicative of higher cumulative incidence compared to American Indians/Asians/Pacific Islanders as well as black children.

### 3.9. Racial and Geographic Disparities in Mortality

Table 2 illustrates the heterogeneity of race as an exposure effect in pediatric cancer mortality, implying the race of the cancer patients as the function of dying. Although not included in this table, there was an association between race and pediatric cancer mortality. Compared to white children, black children were 41% (RR: 1.41, 95% C.I.: 1.31–1.52) more likely to die following cancer diagnosis during the study period (1973–2014). Compared to those who were insured, those with any Medicare (RR: 1.29, 95% C.I.: 1.21–1.36) and those who were uninsured (RR: 1.33, 95% C.I.: 1.13–1.58) were more at risk of dying from pediatric cancer. Compared to children <1 year, those 1–4 years were 37% less likely to die (RR: 0.63, 95% C.I.: 0.57–0.70).

### 3.10. Geographic Locale

After adjusting for age at diagnosis, sex, geographic locale, and tumor registry (SEER) (Table 3), black children were 37% more likely to die, relative to white children (Adjusted Risk Ratio aRR: 1.37, 99% C.I. 1.23–1.52, *p* < 0.0001). Children in rural areas were at higher risk of mortality (aRR = 1.13, C.I.: 0.80–1.60), closely followed by those in urban areas (aRR: 1.05: C.I.: 0.91–1.20).

## 4. Discussion

While overall incidence and mortality in pediatric cancer has improved, incidence continues to increase, and mortality variability persists by health disparities indicators, namely race. Despite the ongoing research in this field, the contributing factors to these disparities and variabilities are not fully understood, which include primarily health inequities such as insurance coverage as a function of access and utilization of cancer treatment. This current study was proposed to examine the temporal trends in overall pediatric cancer incidence, as well as variability in mortality associated with these malignancies. There are a few relevant findings based on our data from the NCI—SEER (1973–2014), one of the longest pediatric cancer temporal trends ever assessed with the SEER dataset, implying more than 40 years of cumulative incidence [19]. Secondly the temporal trend indicates increasing percent and annual percent change. Thirdly, the cumulative incidence was highest among white children relative to black children or others. Fourthly, incidence varied by sex, with males demonstrating higher cumulative incidence. Fifthly, the mortality experience was poorer among black children compared to white children and others; likewise, males experienced lower survival compared to females.

This study has demonstrated the highest cumulative incidence of pediatric cancer among white children. To our knowledge this is one of the longest, implying more than four decades of cumulative incidence of pediatric cancer ever studied using the SEER dataset [20]. The finding of the highest cumulative incidence of pediatric cancer among white children in our data is supported by previous findings in comparable settings, even with the small sample size in the U.S. population [21,22,23,24]. However, because our findings include all malignancies, it may not be accurate with respect to renal carcinoma in children [25]; whereas the total sample findings may apply in explaining research data in the direction of generalizability, the sub-population analysis may differ from the total population. Therefore, by using stratified analysis, sub-population information may become apparent. Given this epidemiological illustration, the findings of increased cumulative incidence of overall cancer among white children may not hold in all malignancies but in most malignancies affecting children in the SEER registry, namely ALL, brain/CNS, lymphoma, and AML.

Our study has shown that males were more likely to be diagnosed with pediatric cancer as well as present with higher mortality rates. These findings are comparable to previously documented epidemiologic and clinical data [1,4,12,26]. Holmes et al. observed an increase in cumulative incidence of leukemia among males compared to females using the same SEER data [27]. Other studies as well have observed similar findings that implicate higher cumulative incidence among males relative to females [5,28,29]. A study by Holmes et al. [27] attempted to explain the excess cumulative incidence among males by variability and hormonal differences. In terms of mortality variability or more males dying from cancer, Holmes et al. indicated the increased diagnosis of T-cell leukemia, which is a more aggressive form of leukemia among males compared to females. Their findings also indicated increased diagnosis of T-cell leukemia, which is the less aggressive form of leukemia among females compared to males. Invariably, it appears that the survival disadvantage of pediatric cancer among males may be attributable to the biologic aggressivity of cancer in general. The biologic plausibility of the observed findings by Holmes et al. may be further explained by epi-genomics comparing the DNA microarray between males and females once diagnosed with pediatric malignancies and followed for the disease. This gene expression is highly likely to indicate the mRNA translation to proteins and metabolic pathway involved in the mutation. It is also plausible that the higher death rates of males may be influenced by the socio epi-genomic variability, implying social determinants and epi-genetic interaction. The further understanding of the sex variance in pediatric cancer requires our ability to understand epi-genomics and its application in the therapeutics (genomic engineering).

The current study has demonstrated the increased mortality rate among black males compared to white in this pediatric sample. While white children continue to illustrate excess cumulative incidence of pediatric cancer, black children perpetually illustrate higher death rates. Our findings of excess mortality among black children are supported by previous epidemiologic and clinical data [19,30]. Holmes et al. did observe the survival disadvantage of black compared to white children in their SEER findings. The authors explained this observation by examining the frequency of T-cell leukemia comparing black and white children with leukemia. Their findings indicated an increasing frequency of T-cell leukemia among black children, implying poor prognosis. This observation in part provided explanation for the excess mortality among black children with respect to leukemia [27]. Since leukemia represents the most diagnosed cancer among children, it is plausible that excess mortality among black children may be driven by the biologic aggressivity of T-cell leukemia. As observed in sex variability, the findings of racial variability require genomic and epi-genomic studies in further understanding the racial variability with the intent to develop therapeutics in addressing this survival variance.

Despite the strength of this study, there are a few limitations. There were unmeasured confoundings such as income, education, and other social determinants that were not available for controlling in the multivariable model in this study. However, it is highly unlikely that the findings of excess mortality among black children are driven solely by unmeasured confoundings.

It is also possible that residual confounding may explain in part the excess mortality or survival disadvantage among black children, since no matter how sophisticated the software used, residual confounding persists [31].

## 5. Conclusions

In summary, this more than four decades of data on cumulative incidence of overall pediatric cancer is indicative of increased incidence among white children and survival disadvantage among black children and males. The understanding of this variability requires further studies on epi-genomic and socio epidemiologic areas in preventing pediatric malignancy and advancing therapeutics.

What is already known on this subject:

- Cancer incidence over the past three decades have been well documented, implying higher incidence among white relative to black children.
- Survival in overall cancer among children has improved, with advantage observed in white children.
- Mortality is influenced by access and utilization of care, namely insurance coverage.

What this study adds:

- Specific cancer incidence such as renal is more common among black children, as well as increasing cancer incidence among white children following a four-decade cancer cumulative incidence assessment.
- Survival disparities by race and sex while lower among black children and males are unexplained by inequity in the distribution of social determinants of health.
- While urbanization (geography) has been observed in adult malignancy, the current study indicates survival disadvantage by rural compared to metropolitan.

## Figures and Tables

**Table 1:** Study Characteristics of Children with Cancer, SEER (1973–2014).

Variable	Black	White	Other	*p*-Value
	N (%)	N (%)	N (%)	
Age at Diagnosis				
<1 year	259 (12.8)	1549 (6.10)	218 (7.7)	<0.001
01–04 years	883 (11.5)	6113 (24.0)	685 (24.3)	
05–09 years	689 (18.6)	4458 (17.5)	482 (17.1)	
10–14 years	797 (21.7)	4943 (19.4)	535 (19.0)	
15–19 years	1076 (29.0)	8383 (32.9)	900 (31.9)	
Sex				
Female	1770 (47.8)	11,770 (46.2)	1283 (45.5)	0.13
Male	1934 (52.2)	13,676 (53.7)	1537 (54.5)	
Insurance				
Any Medicare	1786 (48.2)	8393 (33.0)	787 (27.9)	<0.001
Insured	1444 (39.0)	13,811 (54.3)	1766 (62.6)	
Insured/NS	364 (9.8)	2682 (10.5)	218 (7.7)	
Uninsured	110 (3.0)	560 (2.2)	49 (1.7)	
Location				
Metropolitan	3392 (91.6)	22,905 (90.0)	2652 (94.0)	<0.001
Rural	15 (0.4)	328 (1.3)	1 (0.1)	
Urban	297 (8.0)	2213 (8.7)	167 (5.9)	
Mortality				
Alive	3020 (81.5)	22,134 (87.0)	2390 (84.7)	<0.001
Dead	684 (18.5)	3312 (13.0)	430 (15.2)	
First Malignancy				
Yes	3626 (97.9)	24,933 (98.0)	2759 (97.8)	0.83
No	78 (2.1)	513 (2.0)	61 (2.1)	
SEER Registry				
Atlanta	483 (13.0)	574 (2.2)	83 (2.9)	<0.001
* California	448 (12.1)	7112 (27.9)	706 (25.0)	
Connecticut	147 (4.0)	1078 (4.2)	47 (1.7)	
Detroit	352 (9.5)	1055 (4.1)	49 (1.7)	
Greater Georgia	606 (16.3)	1610 (6.33)	36 (1.3)	
Hawaii	10 (0.3)	93 (0.4)	291 (10.3)	
Iowa	47 (1.3)	1115 (4.4)	28 (1.0)	
Kentucky	132 (3.5)	1411 (5.55)	18 (0.6)	
Los Angeles	272 (7.3)	3029 (11.9)	363 (12.9)	
Louisiana	488 (13.2)	961 (3.8)	37 (1.3)	
New Jersey	431 (11.6)	2577 (10.1)	240 (8.5)	
New Mexico	17 (0.5)	542 (2.1)	79 (2.8)	
Rural Georgia	14 (0.4)	18 (0.0)	0 (0.00)	
San Francisco-Oakland	113 (3.0)	943 (3.7)	336 (11.9)	
San Jose-Monterey	22 (0.6)	766 (3.0)	245 (8.7)	
Seattle	103 (2.8)	1318 (5.2)	214 (7.6)	
Utah	19 (0.5)	1244 (4.9)	48 (1.7)	

Notes: * California excluding San Francisco, San Jose-Monterey and Los Angeles, NS = No Specifics, Other = American Indian/Alaskan Native, Asian/Pacific Islander, Race Unknown = 1158 (1.25%).

**Table 2:** Racial heterogeneity as exposure effect in pediatric cancer mortality.

Variable	RR	95% C.I.	*p*-Value
Race			
White	1.0	Referent	Referent
Black	1.41	1.31–1.52	<0.001
Other	1.17	1.06–1.28	0.001
Age at Diagnosis			
00 years	1.0	Referent	Referent
01–04 years	0.63	0.57–0.70	<0.001
05–09 years	0.72	0.64–0.80	<0.001
10–14 years	0.74	0.67–0.83	<0.001
15–19 years	0.70	0.63–0.77	<0.001
Sex			
Female	1.0	Referent	Referent
Male	1.16	1.10–1.23	<0.001
Insurance			
Insured	1.0	Referent	Referent
Any Medicare	1.29	1.21–1.36	<0.001
Insured/NS	1.02	0.92–1.12	0.64
Uninsured	1.33	1.13–1.58	0.001
Location			
Metropolitan	1.0	Referent	Referent
Rural	1.09	0.85–1.41	0.46
Urban	1.07	0.97–1.18	0.14
SEER Registry			
^†^ Atlanta	1.0	Referent	Referent
* California	0.98	0.84–1.14	0.84
Connecticut	0.77	0.62–0.95	0.01
^†^ Detroit	0.95	0.78–1.15	0.61
Greater Georgia	1.10	0.93–1.31	0.25
Hawaii	1.03	0.78–1.15	0.61
Iowa	0.85	0.69–1.04	0.13
Kentucky	0.94	0.7–1.13	0.50
Los Angeles	1.08	0.92–1.27	0.32
Louisiana	1.18	0.98–1.41	0.07
New Jersey	0.85	0.71–1.00	0.06
New Mexico	1.15	0.92–1.45	0.20
Rural Georgia	1.31	0.63–2.75	0.46
San Francisco-Oakland	0.94	0.77–1.14	0.53
San Jose-Monterey	0.95	0.77–1.17	0.65
Seattle	0.85	0.70–1.03	0.10
Utah	0.88	0.72–1.08	0.23

Abbreviations: RR—Risk ratio, C.I.—Confidence Interval, NS—No Specifics. Notes: * California excluding San Francisco, San Jose-Monterey, and Los Angeles. Other—American Indian/Alaskan Native, Asian/Pacific Islander. ^†^—Metropolitan.

**Table 3:** Racial Disparities in Pediatric Cancer Mortality, SEER (1973–2014).

Variable	aRR	99% C.I.	*p*-Value
Race			
White	1.0	Referent	Referent
Black	1.37	1.23–1.52	<0.001
Other	1.18	1.03–1.34	0.001
Age at Diagnosis			
00 years	1.0	Referent	Referent
01–04 years	0.64	0.55–0.73	<0.001
05–09 years	0.72	0.63–0.84	<0.001
10–14 years	0.75	0.65–0.87	<0.001
15–19 years	0.72	0.63–0.83	<0.001
Sex			
Female	1.0	Referent	Referent
Male	1.16	1.07–1.25	<0.001
Insurance			
Insured	1.0	Referent	Referent
Any Medicare	1.22	1.13–1.32	<0.001
Insured/NS	1.03	0.91–1.18	0.48
Uninsured	1.30	1.04–1.63	0.002
Location			
Metropolitan	1.0	Referent	Referent
Rural	1.13	0.80–1.60	0.37
Urban	1.05	0.91–1.20	0.37
SEER Registry			
Atlanta	1.0	Referent	Referent
* California	1.13	0.92–1.39	0.11
Connecticut	0.89	0.67–1.18	0.29
Detroit	1.04	0.81–1.35	0.64
Greater Georgia	1.16	0.92–1.46	0.08
Hawaii	1.07	0.73–1.56	0.63
Iowa	0.99	0.74–1.31	0.93
Kentucky	1.04	0.80–1.35	0.67
Los Angeles	1.21	0.97–1.50	0.02
Louisiana	1.22	0.96–1.55	0.03
New Jersey	1.00	0.80–1.26	0.93
New Mexico	1.28	0.94–1.73	0.03
Rural Georgia	1.27	0.48–3.36	0.52
San Francisco-Oakland	1.09	0.84–1.41	0.39
San Jose-Monterey	1.10	0.83–1.46	0.35
Seattle	0.99	0.76–1.28	0.93
Utah	1.06	0.81–1.40	0.53

Abbreviations: aRR—Adjusted Risk ratio, C.I.—Confidence Interval, NS—No Specifics. Notes: * California excluding San Francisco, San Jose-Monterey, and Los Angeles, Other—American Indian/Alaskan Native, Asian/Pacific Islander.

## Data Availability

This study utilized secondary data from SEER, NCI.

## Appendix A

**Table A1:** 5-year Cumulative Incidence of Pediatric Cancer for Males by All races (White, Black, Other).

Year	All Races			
	Rate	SE/*p*-Value	Lower CI	Upper CI
1973–1977	14.6	0.6	13.3	15.9
1978–1982	15.0	0.7	13.8	16.4
1983–1987	16.3	0.7	14.9	17.7
1988–1992	16.9	0.7	15.5	18.3
1993–1997	16.9	0.7	15.6	18.3
1998–2002	17.3	0.7	16.0	18.7
2003–2007	17.4	0.7	16.2	18.2
2008–2012	18.5	0.7	17.2	19.9
2013–2014	18.1	0.7	16.8	19.5

**Table A2:** 5-year cumulative incidence of pediatric cancer for males by white children.

Year	Whites			
	Rate	SE/*p*-Value	Lower CI	Upper CI
1973–1977	15.2	0.7	13.8	16.8
1978–1982	15.7	0.7	14.3	17.2
1983–1987	17.3	0.8	15.7	18.9
1988–1992	17.7	0.8	16.1	19.3
1993–1997	17.6	0.8	16.1	19.2
1998–2002	18.6	0.8	17.1	20.3
2003–2007	18.9	0.8	17.3	20.6
2008–2012	19.6	0.8	18.0	21.3
2013–2014	18.8	0.8	17.2	20.4

**Table A3:** 5-year cumulative incidence of pediatric cancer for males by black children.

Year	Black			
	Rate	SE/*p*-Value	Lower CI	Upper CI
1973–1977	10.4	1.7	7.4	14.3
1978–1982	12.1	1.7	8.9	15.9
1983–1987	11.6	1.6	8.6	15.3
1988–1992	13.1	1.7	10.0	16.9
1993–1997	13.2	1.6	10.2	16.7
1998–2002	12.0	1.4	9.3	15.2
2003–2007	12.7	1.5	10.0	15.9
2008–2012	14.7	1.5	11.8	18.0
2013–2014	14.0	1.5	11.2	17.2

**Table A4:** 5-year cumulative incidence for males by other (American Indian/Alaskan Native, Asian/Pacific Islander).

Year	Other			
	Rate	SE/*p*-Value	Lower CI	Upper CI
1973–1977	11.5	2.3	7.4	17.0
1978–1982	11.8	2.1	8.0	16.7
1983–1987	12.8	2.0	9.1	17.6
1988–1992	15.2	2.0	11.3	19.9
1993–1997	15.7	1.9	12.1	20.1
1998–2002	14.4	1.7	11.1	18.3
2003–2007	13.4	1.6	10.4	17.1
2008–2012	14.6	1.6	11.6	18.2
2013–2014	16.9	1.7	13.7	20.7

## Appendix B

**Table A5:** 5-year Cumulative Incidence of Pediatric Cancer for Females by All races (White, Black, Other).

Year	All Races			
	Rate	SE/*p*-Value	Lower CI	Upper CI
1973–1977	13.1	0.6	11.9	14.0
1978–1982	13.8	0.6	12.6	15.1
1983–1987	14.7	0.7	13.4	16.0
1988–1992	15.0	0.7	13.7	16.4
1993–1997	14.8	0.6	13.6	16.2
1998–2002	16.0	0.7	14.8	17.3
2003–2007	15.6	0.6	14.4	17.0
2008–2012	17.2	0.7	15.9	18.5
2013–2014	17.7	0.7	16.3	19.0

**Table A6:** 5-year cumulative incidence of pediatric cancer for females by white children.

Year	White			
	Rate	SE/*p*-Value	Lower CI	Upper CI
1973–1977	13.5	0.7	12.2	15.0
1978–1982	14.3	0.7	12.9	15.7
1983–1987	15.4	0.8	13.9	17.0
1988–1992	15.6	0.8	14.1	17.2
1993–1997	15.6	0.8	14.1	17.2
1998–2002	17.4	0.8	15.9	19.1
2003–2007	16.6	0.8	15.0	18.2
2008–2012	18.2	0.8	16.6	19.9
2013–2014	10.4	0.8	17.4	20.7

**Table A7:** 5-year cumulative incidence of pediatric cancer for females by black children.

Year	Blacks			
	Rate	SE/*p*-Value	Lower CI	Upper CI
1973–1977	11.3	1.8	8.1	15.3
1978–1982	11.9	1.7	8.8	15.7
1983–1987	11.7	1.7	8.7	15.5
1988–1992	12.8	1.7	9.8	16.6
1993–1997	11.6	1.5	8.8	15.0
1998–2002	11.6	1.4	8.9	14.8
2003–2007	12.7	1.5	10.0	15.9
2008–2012	13.9	1.5	11.1	17.3
2013–2014	12.5	1.5	9.8	15.6

**Table A8:** 5-year cumulative incidence for females by other (American Indian/Alaskan Native, Asian/Pacific Islander).

Year	Other			
	Rate	SE/*p*-Value	Lower CI	Upper CI
1973–1977	9.5	2.1	5.9	14.7
1978–1982	11.4	2.1	7.4	16.1
1983–1987	11.2	1.9	7.7	15.8
1988–1992	12.4	1.9	9.0	16.9
1993–1997	12.3	1.8	9.0	16.3
1998–2002	11.8	1.6	8.8	15.5
2003–2007	12	1.6	9.6	16.2
2008–2012	13.4	1.6	10.4	16.9
2013–2014	13.2	1.5	10.3	16.6

## References

[1] Siegel R., Miller K., Jemal A. (2015). Cancer statistics, 2015. CA Cancer J. Clin..

[2] National Center for Health Statistics National Vital Statistics Sysytem. Center for Disease Control. http://www.cdc.gov/injury/wisqars/leadingcauses.html.

[3] The American Cancer Society Medical & Editorial Content Team What Are Childhood Cancers. https://www.cancer.org/cancer/childhood-cancer.html.

[4] Center for Disease Control & Prevention (2007). Trends in Childhood Cancer Mortality—United States, 1990–2004.

[5] Hunger S.P., Mullighan C.G. (2015). Acute Lymphoblastic Leukemia in Children. N. Engl. J. Med..

[6] Belson M., Kingsley B., Holmes A. (2007). Risk Factors for Acute Leukemia in Children: A Review. Environ. Health Perspect..

[7] Margolin J.F., Steuber C.P.P.D. (2001). Acute Lymphocytic Leukemia. Principles and Practice of Pediatric Oncology.

[8] American Cancer Society Medical and Editorial Content Team What Are the Key Statistics for Childhood Leukemia. https://www.cancer.org/cancer/leukemia-in-children/about/key-statistics.html.

[9] The American Cancer Society Medical & Editorial Content Team What Are the Key Statistics about Brain and Spinal Cord Tumors in Children. https://www.cancer.org/cancer/brain-spinal-cord-tumors-children/about/key-statistics.html.

[10] Rickert C.P.W. (2001). Epidemiology of central nervous system tumors in childhood and adolescence based on the new WHO classification. Child’s Nerv Syst..

[11] National Cancer Institute Wilms Tumor and Other Childhood Kidney Tumors Treatment. https://www.cancer.gov/types/kidney/hp/wilms-treatment-pdq#_436.

[12] Holmes L., Vandenberg J., McClarinl L., Dabney K. (2015). Epidemiologic, racial and healthographic mapping of delaware pediatric cancer: 2004–2014. Int. J. Environ. Res. Public Health.

[13] (2023). RMH Patient Information: Prostate Cancer Awareness. https://www.royalmarsden.nhs.uk/private-care/news-and-blogs/prostate-cancer-awareness-all-you-need-know.

[14] The American Cancer Society Medical & Editorial Content Team What Are the Differences Between Cancers in Adults and Children. https://www.cancer.org/cancer/cancer-in-children/differences-adults-children.html.

[15] Program SE and ER (SEER) SEER*Stat Database: Incidence-SEER 9 Regs Research Datac (1973–2014) <Katrina/Rita Population Adjustment>—County Attributes—Total U.S., 1969–2015 Counties. National Cancer Institute. https://seer.cancer.gov/data/citation.html.

[16] Steliarova-Foucher E., Stiller C., Lacour B., Kaatsch P. (2005). International classification of childhood cancer, third edition. Cancer.

[17] Surveillance Research Program, National Cance Institute (2016). SEER*Stat Software.

[18] Hayat M.J., Howlader N., Reichman M.E., Edwards B.K. (2007). Cancer Statistics, Trends, and Multiple Primary Cancer Analyses from the Surveillance, Epidemiology, and End Results (SEER) Program. Oncologist.

[19] Sultan I., Alfaar A.S., Sultan Y., Salman Z., Qaddoumi I. (2025). Trends in childhood cancer: Incidence and survival analysis over 45 years of SEER data. PLoS ONE.

[20] Steliarova-Foucher E., Colombet M., Ries L.A.G., Moreno F., Dolya A., Bray F., Hesseling P., Shin H.Y., Stiller C.A., IICC-3 Contributors (2017). International incidence of childhood cancer, 2001–2010: A population-based registry study. Lancet Oncol..

[21] Ward E., Desantis C., Robbins A., Kohler B., Jemal A. (2014). Childhood and Adolescent Cancer Statistics, 2014. CA Cancer J. Clin..

[22] Linabery A.M., Ross J.A. (2008). Trends in childhood cancer incidence in the U.S. (1992–2004). Cancer.

[23] Kohler B.A., Sherman R.L., Howlader N., Jemal A., Ryerson B.A., Henry K.A., Boscoe F.P., Cronin K.A., Lake A., Noone A.M. (2015). Annual Report to the Nation on the Status of Cancer, 1975–2011, Featuring Incidence of Breast Cancer Subtypes by Race/Ethnicity, Poverty, and State. J. Natl. Cancer Inst..

[24] Erdmann F., Kielkowski D., Schonfeld S.J., Kellett P., Stanulla M., Dickens C., Kaatsch P., Singh E., Schüz J. (2015). Childhood cancer incidence patterns by race, sex and age for 2000–2006: A report from the South African National Cancer Registry. Int. J. Cancer.

[25] Indolfi P., Terenziani M., Casale F., Carli M., Bisogno G., Schiavetti A., Mancini A., Rondelli R., Pession A., Jenkner A. (2003). DTM Renal Cell Carcinoma in Children: A Clinicopathologic Study. J. Clin. Oncol..

[26] Armstrong G.T., Liu Q., Yasui Y., Neglia J.P., Leisenring W., Robison L.L., Mertens A.C. (2009). Late mortality among 5-year survivors of childhood cancer: A summary from the childhood cancer survivor study. J. Clin. Oncol..

[27] Holmes L., Hossain J., Desvignes-Kendrick M., Opara F. (2012). Sex variability in pediatric leukemia survival: Large cohort evidence. ISRN Oncol..

[28] Gurney J.G., Severson R.K., Scott D.R.L. (1995). Cancer in Children in the United States. Cancer.

[29] Baade P.D., Youlden D.R., Valery P.C., Hassall T., Ward L., Green A.C., Aitken J.F. (2010). Trends in incidence of childhood cancer in Australia, 1983–2006. Br. J. Cancer.

[30] Amin R., Bohnert A., Holmes L., Rajasekaran A.A.C. (2010). Epidemiologic Mapping of Florida Childhood Cancer Clusters. Pediatr. Blood Cancer.

[31] Holmes L., Chan W., Jiang Z.D.X. (2007). Effectiveness of androgen deprivation therapy in prolonging survival of older mean treated for locoregional prostate cancer. Prostate Cancer Prostatic Dis..

